# Protective effects of carvacrol on lipid profiles, oxidative stress, hypertension, and cardiac dysfunction – A comprehensive review

**DOI:** 10.1002/fsn3.4014

**Published:** 2024-02-05

**Authors:** Mohammad Reza Khazdair, Mozhgan Moshtagh, Akbar Anaeigoudari, Shima Jafari, Toba Kazemi

**Affiliations:** ^1^ Cardiovascular Diseases Research Center Birjand University of Medical Sciences Birjand Iran; ^2^ Social Determinants of Health Research Center Birjand University of Medical Sciences Birjand Iran; ^3^ Department of Physiology, School of Medicine Jiroft University of Medical Sciences Jiroft Iran; ^4^ Department of Clinical Pharmacy, School of Pharmacy Birjand University of Medical Sciences Birjand Iran

**Keywords:** cardiac dysfunction, carvacrol, hypertension, lipid profile, oxidative stress

## Abstract

Cardiovascular diseases (CVDs) are a class of illnesses that affect the heart or blood vessels, leading to the most common causes of death worldwide. In 2017, CVD caused approximately 17.8 million deaths that were increased approximately to 20.5 million deaths in 2021, globally. Also, nearly 80% of worldwide CVD deaths occur in some countries. Some herbs and their constituents due to their several pharmacological activities have been used for medicinal purposes. Carvacrol is a phenolic mono‐terpenoid found in the oils of aromatic herbs with several biological properties. The possible therapeutic effects of carvacrol on lipid profiles, oxidative stress, hypertension, and cardiac dysfunction were summarized in the current study. The data from this review article were obtained by searching the terms including; “Carvacrol”, “Hypertension”, Hypotensive, “Cardiac dysfunction”, “Ischemia”, “Lipid profile”, and Oxidative stress in several web databases such as Web of Sciences, PubMed Central, and Google Scholar, until November 2023. The results of the reviewed studies revealed that carvacrol inhibits acetylcholinesterase (AchE) activity and alters lipid profiles, reducing heart rate as well as systolic and diastolic blood pressure (BP). Carvacrol also decreased the proinflammatory cytokine (IL‐1β), while increasing secretion of anti‐inflammatory cytokine (IL‐10). Moreover, carvacrol improved oxidative stress and mitigated the number of apoptotic cells. The pharmacological effects of carvacrol on CVD might be through its antioxidative, anti‐inflammatory, and antiapoptotic effects. The mentioned therapeutic effects of carvacrol on lipid profile, hypertension, and cardiac dysfunction indicate the possible remedy effect of carvacrol for the treatment of CVD.

## Graphical abstract

The medicinal plants and their constituents due to their several pharmacological activities have been used for medicinal purposes. Carvacrol is a phenolic mono‐terpenoid found in the oils of aromatic plants and has several biological properties. Carvacrol inhibits acetylcholinesterase (AchE) activity and alters lipid profiles, reducing heart rate and systolic and diastolic blood pressure. Administration of carvacrol also decreased the proinflammatory cytokine (IL‐1β), while increasing production of anti‐inflammatory cytokine (IL‐10). Moreover, carvacrol improved oxidative stress and mitigated the number of apoptotic cells.

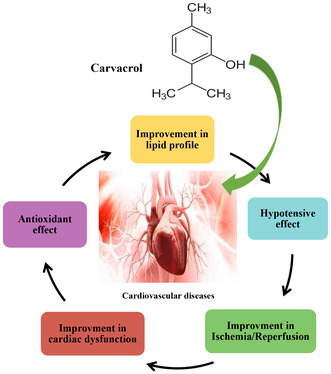

## 1 INTRODUCTION

Cardiovascular diseases (CVDs) are the leading causes of death worldwide according to the World Health Organization (WHO) reports (Benjamin et al., 2017). CVDs cover a wide range of illnesses, including stroke, heart failure, acute coronary syndrome, coronary heart disease, peripheral vascular diseases, hypertension, and dyslipidemias (Dias et al., 2022).

Risk factors for CVDs include diabetes, dyslipidemia, obesity, high blood pressure, smoking, and a sedentary (Ahmad et al., 1999) lifestyle (Shaito et al., 2020). By identifying and avoiding these risk factors, CVD prevalence can be reduced. CVD risk factors are divided into two classes: modifiable and nonmodifiable risk factors. The main regimens for preventing and/or treating CVDs include lifestyle adjustments, lipid‐lowering medications, antihypertensive, antiplatelet, and anticoagulant treatments (Flora & Nayak, 2019).

The global trends of total CVD prevalence death increased to nearly 18.6 million in 2019 (Roth et al., 2020). In Iran, CVD is the most common cause of death (46%) and the disability‐adjusted life years (DALYs) (Sarrafzadegan & Mohammmadifard, 2019).

The trends of CVD‐related deaths during the COVID‐19 pandemic showed significant downward in hypertensive diseases (20%–16.4% in March 2020–June 2021, respectively), while other CVD‐related diseases such as cardiac arrhythmias and heart failure showed significant upward (Vasudeva et al., 2022). Furthermore, CVD also was the most common cause of death even during the COVID‐19 pandemic in the Iranian population (Pirayesh et al., 2023).

Plants and natural products have been utilized for hundreds of years for therapeutic purposes and as a source for developing new medications that help in disease treatment. The consumer preference for natural remedies, as well as the adverse effects and costly expense of synthetic medications, fueled interest (Calixto, 2000). Natural substances such as terpenes show beneficial pharmacological properties, boost the antioxidant systems, and modify redox signaling. Ameliorated effects of genistein, a polyphenolic isoflavone, on the cardiovascular system, including improvement in cardiac dysfunction, lowering blood pressure (BP), modulating lipid profile, and improvement ischemia–reperfusion of the heart, have been reported (Jafari et al., 2023). Terpene chemicals are secondary metabolites that are found largely in herbs as ingredients of essential oils (Abdul Ghani et al., 2023). There are just two terpene phenolic chemicals present in essential oils: thymol and carvacrol (Abdul Ghani et al., 2023).

Carvacrol is a mono‐terpenoid molecule found in the oils of aromatic plant species such as thymus, origanum, and pepperwort (Al Seyedan et al., 2020). It is also most common in *Nigella sativa* L., of the Ranunculaceae family. The immunomodulatory of medicinal herbs and main constituents such as carvacrol was reported (Khazdair et al., 2021). The genus Origanum is widely used for various purposes such as food additive and medicine in traditional medicine (Khazdair et al., 2021). Carvacrol is the main ingredient of *Zataria multiflora*, a well‐known medicinal plant that has been used as a spices in culinary and is used as herbal medicine for the treatment of different diseases in Khorasan province (Khazdair, Ghorani, et al., 2018).

Carvacrol has several biological activities, including antifungal and antibacterial, antiviral, antioxidant, and anticarcinogenic effects (Sharifi‐Rad et al., 2018). Therapeutic effects of carvacrol such as its antioxidant and anti‐inflammatory properties in patients with lung disorders were also reported (Khazdair, Alavinezhad, et al., 2018; Khazdair & Boskabady, 2019).

Previous research has linked dietary consumption of carvacrol to a lower risk of cardiovascular disease; consequently, it appears that the time has come to review the evidence of varied effects of carvacrol on CVDs. Therefore, investigating the potential effects of carvacrol for the prevention or treatment of CVDs, including (1) Effects on lipid profile and oxidative stress, (2) Hypotensive effects, and (3) Effects on cardiac dysfunction and Ischemia/Reperfusion, is the purpose of the current review study.

## 2 METHOD

Information of the present review study was achieved via searching the following keywords, including “Carvacrol”, “Hypertension”, Hypotensive, “Cardiac dysfunction”, “Ischemia”, “Lipid profile”, and Oxidative stress in various accessible web databases, including Web of Sciences, PubMed Central, and Google Scholar until November 2023.

Firstly, original articles were obtained by two researchers (MK and MM) with doctoral degrees. Then, they carefully evaluated the papers regarding their suitability for selecting to review.

**FIGURE 1:**
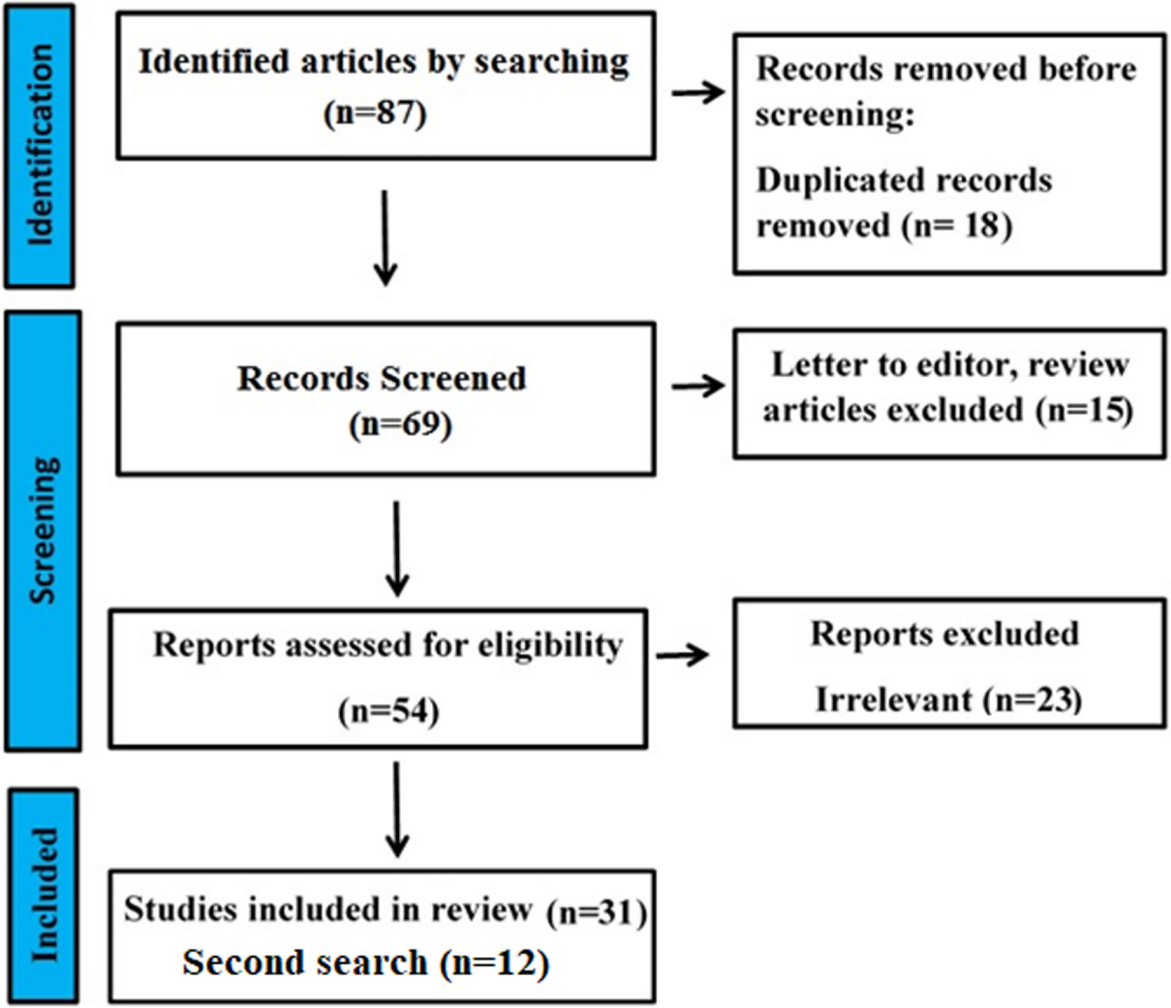
Flowchart for literature screening.

Letter to the editor and published papers as full in other than English were excluded. Among 94 articles obtained from the different databases, 18 studies were duplicates, and 39 articles, including reviews and papers without suitable data or enough details, were removed to decrease their effects on the quality of results. Finally, according to the flowchart of included studies (Figure 1), 31 papers at the preparation stage of the manuscript and 12 papers in the requested revision stage, related to the subject of this study were included.

## 3 RESULTS

### 3.1 Effects on lipid profile and oxidative stress

#### 3.1.1 In vitro studies

The inhibitory effects of carvacrol, thymol, eugenol, and piperine (20–50 mg/L) in yeasts were evaluated. It was reported that those amounts can be influenced by total lipids, accumulation amount of triacylglycerol, and the average of lipid body size. However, these effects were not the same at higher doses (50 mg/L). It appears that the total carbon entry into the triacylglycerol biosynthesis is curbed, thus its accumulation would be reduced (Kimura et al., 2006).

Anti‐inflammatory and antiobesity effects of carvacrol derivatives (CD1‐3) were investigated in vitro (3T3‐L1, WJ‐MSCs, and THP‐1 cell lines) by assessing the expression of obesity‐related proteins and reducing TNF‐α expression, respectively. A direct linkage was found between carvacrol and naproxen displaying antiobesity (inhibition of lipids accumulation) and anti‐inflammatory properties of carvacrol derivatives in vitro (Cacciatore et al., 2023).

The protective and lipid‐lowering effects of carvacrol in hepatic steatosis (cellular models) and endothelial dysfunction indicated that exposure of cells (endothelial HECV cells) with carvacrol counteracted lipid accumulation in hepatocytes and secured endothelial cells from dysfunction and oxidative stress (Khalil et al., 2022). Treatment with carvacrol (0.03–3 μM) remarkably inhibited platelet‐derived growth factor (PDGF)‐BB‐stimulated rat aortic smooth muscle cells proliferation and migration. Carvacrol reduced the expression of NADPH oxidase (NOX) 1 and the phosphorylation of MAPK (Lee et al., 2015).

#### 3.1.2 In vivo studies

The hypolipidemic effects of carvacrol fed in Wistar rats and inhibiting acetylcholine have been observed in a study. According to this research, malathion and parathion (PTN) as toxic pesticides, significantly increased cholesterol and triglyceride, while administration of 25 mg/kg of carvacrol with parathion could inhibit acetylcholinesterase (AchE) activity and alter lipid profiles (Salari et al., 2021). Daily carvacrol feeding (50 mg/kg/day) singly or combined with simvastatin (20 mg/kg/day) in rats with hyperlipidemia induced by injecting poloxamer 407 (500 mg/kg) resulted in hypolipidemic effects including decreasing total cholesterol (TC), triglycerides, low‐density lipoprotein (LDL), leptin, atherogenic index, and increasing adiponectin and high‐density lipoprotein (HDL). Furthermore, combinational treatment with carvacrol modified liver and muscle injury induced by simvastatin (Abd El Aal et al., 2017).

Oral carvacrol unveiled different effects on blood lipids and sex hormones in broiler chickens depending upon the doses used. A higher dose (0.5 g/L) remarkably decreased the percentage of abdominal fat. On the other hand, the birds receiving carvacrol at higher doses (>0.2 g/L) showed an increase in serum testosterone compared to the control birds (Khosravinia, 2014). Administration of carvacrol (0.1%) as supplemented diet decreased body weight (BW) and visceral fat‐pad weights and reduced levels of plasma lipid in high‐fat diet mice. It seems that visceral adipogenesis is inhibited owing to suppressing bone morphogenic protein, galanin‐mediated signaling, and fibroblast growth factor 1 (FGF‐1) as well as reducing the production of proinflammatory mediators in visceral adipose tissues (Cho et al., 2012). Combinational use of thymol (100) and carvacrol (200 mg/kg) in birds fed diets supplemented with carboxyl methyl cellulose manifested the decrease in digesta viscosity and serum TC. Combining thymol and carvacrol was associated with a remarkable increase in albumin, globulin, and total protein (Hashemipour et al., 2014).

Carvacrol injection (0.6 mL/94% purity) in broiler chicks significantly increased the blood calcium concentration. However, no effect was found on the absorption of cholesterol from yolk sac residuals. The slope of decreasing in plasma glucose amount was slower in treated birds with carvacrol, and cholesterol levels were lower compared to the other treatments at 72 h posthatch (Beiranvand et al., 2016). Carvacrol (0.1% (w/w)) consumption in a study on mice fed with a high‐fat diet (HFD) significantly decreased levels of hepatic lipids. It also reduced the activities of aspartate aminotransferase (AST), plasma alanine aminotransferase (ALT), amounts of monocyte chemoattractant protein 1 (MCP‐1), and tumor necrosis factor‐alpha (TNF‐α). Based on this study's findings, carvacrol can prevent HFD‐induced hepatic steatosis (Kim et al., 2013). Dietary carvacrol (200 ppm) in broilers resulted in reducing plasma triglyceride amounts, but no effect was found on plasma cholesterol. According to this study, the effect of thymol and carvacrol on growth efficiency and metabolism of triglyceride are different in broiler chickens (Lee et al., 2003). Carvacrol (150 mg/kg) administration in broiler chickens decreased lipid oxidation or 2‐TBA reactive substances (TBARS). Five to ten days of sample storage was associated with a notable increase in the levels of TBARS, while groups receiving thymol or carvacrol had lower values of TBARS. Thus, thymol or carvacrol can be applied as natural antioxidants to improve the quality of birds' meat (Luna et al., 2010).

The effects of dietary supplementation of thymol and carvacrol were evaluated at 4 levels (0, 60, 100, and 200 mg/kg of diet) in broiler chickens. Although the immune response was improved using thymol + carvacrol, any effects were not observed in blood markers and the weight of lymphoid organs. The activity of antioxidant and digestive enzymes, as well as the immune response of broilers, improved while lipid oxidation decreased as a result of thymol + carvacrol supplementation (Hashemipour et al., 2013). The effects of *Zataria multiflora* (ZM) extract and carvacrol on Adriamycin‐induced cardiotoxicity were investigated in four groups of rats. Blood tests were conducted on days 0 and 28 for Control, ADR group (intravenously single dose), ZM + ADR, and carvacrol + ADR groups (gavage of ZM and carvacrol for 28 days). An increase in superoxide dismutase (SOD) activity, reduction of total thiol contents, as well as SGPT and SGOT levels were significantly modified by carvacrol application. The oxidative stress damage and cardiotoxicity with ADR were improved by getting ZM and carvacrol (Khajavi Rad & Mohebbati, 2018).

The effectiveness of *Zataria multiflora* extract and carvacrol on oxidative stress parameters and nitric oxide (NO) in lipopolysaccharide (LPS) induced aortic and cardiac injury was assessed in Wistar rats. Treatment with carvacrol (25–100 mg/kg) 3 days before and during the intervention enhanced the levels of SOD, CAT, and thiols, while decreasing malondialdehyde (MDA) and NO in the cardiac and aortic tissues (Hosseini et al., 2023).

The protective effects of carvacrol on ketamine‐induced cardiotoxicity were shown by decreasing the levels of MDA, TNF‐α, IL‐1β, and IL‐6 in the tissue and increasing the levels of antioxidant parameters such as in tissue glutathione (GSH) and SOD levels in the heart tissues (Ölmeztürk Karakurt et al., 2022). Carvacrol could mitigate the negative effects of ketamine by preventing lipid peroxidation and inflammatory response. These results indicated that carvacrol could be considered as a medicine to treat alteration in lipid profile and owing to the favorable effects as well as inhibition of oxidative stress, it could be considered as a cardioprotective agent. The effects of carvacrol on lipid profile are shown in Table 1.

**TABLE 1:** The effects of carvacrol on lipid profile and oxidative stress (OS).

Study design	Doses	Route of administration	Effects	Ref.
In vitro (Oleaginous Yeast)	20–50 mg/L	Expose	↓ Total lipid and triacylglycerol accumulation amount, and the average lipid body size	Kimura et al. (2006)
In vitro (cell lines)	25 μM	Expose	Inhibitory effect on lipid accumulation in both 3 T3‐L1 and WJ‐MSCs cells ↓ TNF‐α levels ↓ Expression levels of transcription factor carbohydrate response element binding protein (ChREBP) which is the major factor responsible for de novo lipogenesis	Cacciatore et al. (2023)
Aortic smooth muscle cells	0.03–3 μM		Significantly inhibited platelet‐derived growth factor (PDGF)‐BB‐stimulated rat aortic smooth muscle cell migration and proliferation ↓ The expression of NADPH oxidase (NOX) 1 and the phosphorylation of p38 mitogen‐activated protein kinase (MAPK)	Lee et al. (2015)
Wistar rats	25 mg/kg	Per Os (by mouth/oral) (P.O) 10 days	↓ Serum AchE activity ↓ TG and cholesterol in parathion (PTN) group but not in Malathion (MTN) group	Salari et al. (2021)
Hyperlipidemia in rats	20 mg/kg/day	P.O 30 days	↓ Total cholesterol ↓ TG ↓ LDL ↓ Leptin and atherogenic index ↑ HDL and adiponectin	Abd El Aal et al. (2017)
Broiler chicken	0.3 and 0.5 g/L	P.O Drinking water 28 day	↓ Abdominal fat (E 1) ↓ Serum estradiol (E 2) ↑ Serum testosterone (E 1& E 2)	Khosravinia (2014)
(C57BL/6N) Mice	0.1%‐supplemented diet (CSD)	P.O	↓ Weight, visceral fat‐pad, and plasma lipids (CSD compared with HFD) ↓ Bone morphogenic protein, ↓ Fibroblast growth factor 1, galanin‐mediated signaling ↓ Proinflammatory cytokines in visceral adipose tissues	Cho et al. (2012)
Birds	Thymol (100) + Carvacrol (200 mg/kg)	P.O	↓ Digesta viscosity and serum total cholesterol ↑ Total protein, albumin, and globulin (suppress the total carbon inflow into the triacylglycerol biosynthesis)	Hashemipour et al. (2014)
Broiler chicken	0.6 mL/94% purity	Intraperitoneal injection (I.P)	↑ The blood calcium concentration ↓ Cholesterol levels	Beiranvand et al. (2016)
Mice fed with a HFD	0.1% (w/w)	P.O	↓ Hepatic lipid levels ↓ Activities of plasma ALT, AST, and amounts of MCP‐1 and TNF‐α ↓ HFD‐induced hepatic steatosis	Kim et al. (2013)
Broiler chicken	200 ppm	P.O	↓ Plasma triglyceride amounts ↓ Plasma triglyceride concentration ↓ Weight gain	Lee et al. (2003)
Broiler chicken	150 mg/kg	P.O	↓ Lipid oxidation (values of TBARS)	Luna et al. (2010)
Broiler chicken	0, 60, 100, and 200 mg/kg	P.O	↑ Activity of antioxidant and immune response	Hashemipour et al. (2013)
Rats	‐	P.O	↑ Superoxide dismutase (SOD) activity ↓ Total thiol contents, as well as SGPT and SGOT levels	Khajavi Rad and Mohebbati (2018)
Wistar rats	25–100 mg/kg	P.O	↓ Superoxide dismutase (SOD), catalase (CAT), and thiols ↓ Malondialdehyde (MDA) and nitric oxide (NO) in cardiac and aortic tissues	Hosseini et al. (2023)
Rats	20 mg/kg	P.O	↓ Negative effects of ketamine ↓ Lipid peroxidation and inflammatory response	Ölmeztürk Karakurt et al. (2022)

Abbreviations: AchE, Acetylcholinesterase; ALT, Alanine aminotransferase; AST, Aspartate aminotransferase; CSD, Carvacrol‐supplemented diet; HDL, High‐Density Lipoprotein; HFD, High‐fat diet; I.P, Intraperitoneal injection; LDL, Low‐density lipoprotein; MCP‐1, Monocyte chemoattractant protein 1; P.O, Per Os or by mouth/oral; PTN, Parathion; SGOT, Serum glutamic oxaloacetic transaminase; SGPT, Serum glutamic pyruvic transaminase; TBARS, Thiobarbituric acid reactive substances; TG, Triglycerides; TNF‐α, Tumor necrosis factor α.

### 3.2 Hypotensive effects

#### 3.2.1 In vitro studies

Administration of carvacrol (10^−4^ M) on phenylephrine and calcium chloride (CaCl_2_) induced contractions of isolated rat aorta indicated that carvacrol has no inhibitory action on adrenergic receptor or voltage‐dependent vascular L‐type calcium channels, while its blocking actions on cardiac *L*‐type calcium channel was suggested for the hypotensive actions of carvacrol (Aydin et al., 2007). Vaso‐relaxant effects of carvacrol and thymol in rat‐isolated aorta were assessed. Thymol and carvacrol showed relaxant effects on KCl or phenylephrine precontracted aortic ring tissue. In addition, carvacrol and thymol (400 μM) remarkably reduced the CaCl_2_‐induced contractions and remarkably decreased phorbol dibutyrate‐induced contraction (Peixoto‐Neves et al., 2010). Carvacrol at doses (0.1–100 μM) also produced full vaso‐relaxing effect on 20 mM KCl precontracted rat aortic tissue, both in the presence and in the absence of the endothelial layer. This effects mainly through an inhibition of calcium influx, with the participation of the transient receptor potential (TRP) cation channels (Testai et al., 2016).

The effect of carvacrol on phenylephrine‐induced arterial constriction model showed that carvacrol (25, 50, and 100 μM) induced relaxation of arteries precontracted with phenylephrine. Moreover, carvacrol pretreatment for 20 min resulted in a significant prevention of vasoconstriction induced by phenylephrine (Liu et al., 2020).

The aortic rings were exposed to norepinephrine (NA) (1 μM), α,β‐methylene ATP (1 μM), and potassium chloride (KCl) (60 mM) in the presence of carvacrol (0.01–20 mg/mL) reduced the contractility in the rat thoracic aorta (Shatarat et al., 2023).

#### 3.2.2 In vivo studies

The effects of carvacrol for modulating endothelial progenitor cells (EPC) in hypertensive rats were evaluated. Spontaneously hypertensive rats (SHR) received oral carvacrol (50 or 100 mg/kg/day) or resveratrol (10 mg/kg/day) for 1 month. Evaluating the ROS, CD34, and CD31quantity, EPCs were separated from the bone marrow and peripheral circulation and were measured by flow cytometry. EPC's role was assessed by detecting colony‐forming units (CFU), endothelial nitric oxide synthase (eNOS), and senescence, as well as intracellular reactive oxygen species (ROS). EPC migration, increasing CFU production, expression of eNOS, surface antigens (CD31 and CD34), and reduction in ROS and senescence (aging) were observed as a result of getting carvacrol. In other words, carvacrol, modified endothelial dysfunction, and EPC function (Gonçalves et al., 2023). The action mechanism of calcium channels and transient receptor potential (TRP) channels in normotensive rats by applying intravenous (i.v) administration of carvacrol (1, 5, 10, and 20 mg/kg) was investigated. Carvacrol inhibited contractions induced by phenylephrine and Ca^2+^ influx through L‐type channel. The carvacrol effect was weakened by Mg^2+^ and enhanced by La^3+^ and Gd^3+^, indicating that TRP channels contribute to inducing relaxation by carvacrol. Thus, inhibiting Ca^2+^ influx through Cav and TRP channels, carvacrol takes part in bradycardia, and peripheral vasodilatation that leads to hypotension (Dantas et al., 2015).

Intraperitoneal injection (ip.) of carvacrol (100 μg/kg) reduced heart rate, arterial pressure, as well as systolic and diastolic BP of the anesthetized rats. Carvacrol also exhibited hypotension effect by inhibiting *N*_(omega)_‐nitro‐*L*‐arginine methyl ester (*L*‐NAME) induced hypertension (HR) (Aydin et al., 2007). Consumption of free and encapsulation of carvacrol (50 mg/kg) in β‐cyclodextrin was evaluated in spontaneously hypertensive rats (SHRs). Arterial HR in SHRs receiving carvacrol/β‐CD was remarkably reduced compared with those who got the free form. It is concluded that carvacrol in β‐CD is improving cardiovascular activity (Barreto da Silva et al., 2020).

Treatment with carvacrol (25–100 mg/kg) for 21 days in lead‐exposed rats decreased their BP and inhibited the lead‐induced changes such as decreasing blood parameters (RBC, Hb, Hct, and WBC) compared to control groups. Therefore, carvacrol could have improving effects on the cardiovascular system and mitigate changes induced by lead (Zare Mehrjerdi et al., 2018). The effectiveness of carvacrol on the cardiovascular system of SHR was assessed. Two control groups only got sorbitol, the third one received losartan, and carvacrol (50 and 20 mg/kg, respectively) was administered for the fourth group. All of those drugs are used daily by oral gavage for 1 month. Carvacrol caused a reduction in HR and peripheral vascular resistance, while improving the expression of angiotensin 1–7 receptors, called “Mas” receptors in the kidney (Dias et al., 2022).

Effects of carvacrol combined with aerobic exercise treatment on SHR were evaluated. Six groups including three control ones (normotensive, hypertensive, and receiving amlodipine (20 mg)) compared with tests (carvacrol (20 mg), exercise, and carvacrol in combination with exercise) for 4 weeks. SHR, who get a combined treatment with carvacrol and exercise, revealed a greater reduction in systolic blood pressure and atherogenic indices or improved lipid parameters. Based on the conclusion, exercise was suggested for controlling the biochemical factors of cardiovascular risk (Costa et al., 2021). Carvacrol's effects on the cardiovascular functions of anesthetized rats were investigated. Carvacrol (100 μg/kg) injection in normotensive anesthetized rats exhibited a reduction in heart rate, systolic and diastolic BP, and arterial pressure, while lower doses of carvacrol (1–20 μg/kg) had no effects on blood pressure. Hypotensive effects of carvacrol were concluded to be associated with inhibiting L‐NAME‐induced HR (Aydin et al., 2007). The results of these studies demonstrated that inhibition of voltage‐dependent calcium channels and different transient receptor potential (TRP) channels as well as reduction in hypertrophy markers by carvacrol might be involved in its hypotensive effect. Also, carvacrol as an antagonist of calcium channel receptors could decrease vascular reactivity to different concentrations of calcium and noradrenaline, leading to a reduction in BP. The hypotensive effects of carvacrol are shown in Table 2.

**TABLE 2:** The effects of carvacrol on hypertension.

Study design	Doses	Route of administration	Effects	Ref.
In vitro/Isolated rat aorta	10^−4^ M	Expose	Lack of effect on adrenergic receptors or voltage‐dependent vascular L‐type calcium channels Lack of hypotensive action on isolated rat aorta	Aydin et al. (2007)
Isolated rat aorta	400 μM	Expose	Showed relaxant effects on aortic ring preparations precontracted using KCl or using phenylephrine. Reduced the CaCl_2_‐induced contractions	Peixoto‐Neves et al. (2010)
Isolated rat aorta	0.1–100 μM	Expose	Produced full vaso‐relaxing effect on 20 mM KCl‐precontracted rat aortic preparations.	Testai et al. (2016)
Isolated rat aorta	25, 50, and 100 μM	Expose	Induced relaxation of arteries precontracted with phenylephrine. Moreover, carvacrol pretreatment significantly prevented vasoconstriction induced by phenylephrine.	Liu et al. (2020)
Isolated rat aorta	0.01–20 mg/mL	Expose	↓ The contractility of aortic rings was exposed to norepinephrine (NA), α,β‐methylene ATP, and potassium chloride (KCl).	Shatarat et al. (2023)
Hypertensive rats	50 or 100 mg/kg/day	P.O	↑ Expression of eNOS, surface antigens (CD31 and CD34) ↓ ROS and senescence (aging) and modified endothelial dysfunction	Gonçalves et al. (2023)
Normotensive rats	1, 5, 10, and 20 mg/kg	Intravenous (i.v)	Inhibited contractions induced by phenylephrine and Ca^2+^ influx through L‐type channel.	Dantas et al. (2015)
Rats	100 μg/kg	I.P	↓ Heart rate ↓ Mean Arterial Pressure ↓ Systolic and Diastolic BP (anesthetized rats)	Aydin et al. (2007)
Spontaneously hypertensive rats (SHRs)	50 mg/kg/day	P.O	↓ Arterial hypertension in SHR ↓ Proinflammatory mediator and IL‐1β ↑ Anti‐inflammatory cytokine and IL‐10	Barreto da Silva et al. (2020)
Wistar rats	25, 50, and 100 mg/kg/daily	P.O	↓ Hypertension Preventing lead‐induced changes (RBC, Hb, Hct, and WBC)	Zare Mehrjerdi et al. (2018)
In vivo (SHRs)	20 mg/kg	Oral gavage	Antihypertensive effect ↓ PVR (peripheral vascular resistance) ↑ Expression of MAS receptors in kidney	Dias et al. (2022)
In vivo (SHRs)	20 mg	Oral gavage	↓ Systolic blood pressure ↓ Atherogenic indices Improved lipid parameters	Costa et al. (2021)
Normotensive anesthetized rats	100 μg/kg	I.P	↓ Heart rate ↓ Systolic and diastolic blood pressure ↓ Mean arterial pressure	Aydin et al. (2007)

Abbreviations: BP, Blood pressure; Cav, channel; CFU, Colony‐forming units; eNOS, Endothelial nitric oxide synthase; EPC, Endothelial progenitor cells; Hb, Hemoglobin; Hct, Hematocrit; HR, Heart rate; I.P, Intraperitoneal injection; LDL, Low‐density lipoprotein; P.O, Per Os or by mouth/oral; ROS, Reactive oxygen species; RBC, Red blood cell; SHR, Spontaneously hypertensive rats; TRP, Transient receptor potential; WBC, White blood cell.

### 3.3 Effects on cardiac dysfunctions and ischemia/reperfusion

One of the cardiovascular diseases that happens in patients with diabetes mellitus (DM) is diabetic cardiomyopathy (DCM). This cardiovascular disorder is associated with structural and functional alterations (Jia et al., 2016). In addition, cardiac fibrosis, left ventricular hypertrophy, and diastolic and systolic disturbance resulting from DM have been seen in DCM (Yue et al., 2017). Glucose transporter type 4 (GLUT4) has been shown to supply most of the glucose to the cardiomyocytes. It has been also detected that the expression of GLUT4 reduced in the cardiac muscle in diabetic animals (Stratmann et al., 2022). On the other hand, activation of the insulin‐stimulated phosphatidylinositol 3‐kinase (PI3K)/AKT pathway regulated the expression of GLUT4 (Ramachandran & Saravanan, 2015). It has been also determined that diabetes disturbs the function of this intracellular signaling pathway (Qi & Zhong, 2018).

Angiotensin II (Ang II) was used to induce hypertrophy in an in vitro model study. Based on the results of the study, treatment with carvacrol (0.01 and 0.1 μM) decreased the size of rat cardiomyocyte H9c2 cells. These findings revealed that carvacrol could mitigate the number of apoptotic cells and improve oxidative stress (Jamhiri et al., 2019). The use of 6.25 and 50 μM of carvacrol for 24 h in another in vitro study protected HL‐1 cardiomyocytes exposed by LPS via suppressing TLR4/NFκB/NALP3/IL‐1β inflammatory signaling pathway and inhibiting ROS production (Marconi et al., 2022). In another study, treatment of LPS‐stimulated H9c2 cardiomyoblast cells with carvacrol (2.5, 5, and 10 μg/mL) inhibited ROS generation and abated pyroptosis mediated by NOD‐like receptor family pyrin domain containing 3 (NLRP3) inflammasome in H9c2 cells (Joshi et al., 2023).

In a mice model of type 1 and type 2 DM, peripheral treatment of 20 mg/kg of carvacrol for weeks ameliorated DCM via modulating PI3K/AKT signaling (Hou et al., 2019). The findings of the study of Zhao et al. (2020) demonstrated that oral administration of 5 and 10 mg/kg of carvacrol for 6 weeks improved endothelial dysfunction in diabetic db/db mice by attenuating the inflammation reactions. Left ventricular hypertrophy (LVH) is an adaptive response resulting from chronic hypertension that is associated with increased levels of protein synthesis and extracellular matrix dysfunction (Gupta et al., 2007). LVH has been also understood to be a key contributor causing heart failure and mortality (Shimizu & Minamino, 2016). It has been documented that cardiac malfunction induced by LVH is accompanied by overproduction of free radicals and oxidative stress (Maulik & Kumar, 2012). In an in vivo study, administration of carvacrol (50 and 75 mg/kg for 3 weeks) in rats undergoing abdominal aorta banding revealed that carvacrol could mitigate the level of malondialdehyde (MDA) and expression of atrial natriuretic peptide (ANP) and enhanced the 2‐2‐diphenyl 1‐picril‐hydrasil (DPPH) radical scavenging activity (Jamhiri et al., 2019). Sadeghzadeh et al. (2018) showed that systemic administration of 10, 25, and 50 mg/kg of carvacrol for 4 weeks could restore hypertension and left ventricular hypertrophy (LVH) resulting from abdominal aortic banding in rats. Cardioprotective effect of carvacrol was linked to the antihypertensive antiapoptotic effects of this monoterpene (Sadeghzadeh et al., 2018). Hypertension is a cardiovascular disorder related to inappropriate diet, smoking, gaining, and heredity which enhances the risk of cardiovascular damage and mortality (Mills et al., 2016).

Doxorubicin (DOX) is a type of chemotherapy drug that prevents the growth of tumors via inhibiting topo isomerase 2 (Farina et al., 2021). This anticancer drug also possesses cardiotoxicity properties (Christidi & Brunham, 2021). In a study conducted by Jafarinezhad et al. (2019), oral administration of 50 mg/kg of carvacrol for 6 weeks prevented the harmful effects of DOX on the cardiac tissue of rats. The cardioprotective effect of DOX was accompanied by an improvement of electrocardiogram recording, troponin I content, and ventricular function (Jafarinezhad et al., 2019). In a similar study, the use of 25 mg/kg of carvacrol for 14 days ameliorated cardiotoxicity caused by DOX via modulating oxidative stress and inhibiting inflammation and apoptosis (El‐Sayed et al., 2016). Cyclophosphamide as a cytotoxic substance has immunosuppressive properties and is used for curing various benign tumors (Berköz et al., 2021). The cytotoxic effect of cyclophosphamide is mainly exerted by evoking the production of ROS and inducing oxidative stress (Shalayel et al., 2021). Cardiotoxicity has been also reported as one of the side effects of this drug when it is used in high doses. It has been found that 5 and 10 mg/kg of carvacrol for 6 days could rescue cardiomyocytes from toxicity triggered by cyclophosphamide in rats. The protective effect of cyclophosphamide is associated with a significant decrease in inflammation and lipid peroxidation and a remarkable increase in blood level of total antioxidant capacity (TAS) and glutathione (GSH) in the heart tissue (Cetik et al., 2015). Lipopolysaccharide (LPS) is a bacterial toxin that induces inflammatory reactions and oxidative stress (Beheshti et al., 2022).

Myocardial ischemic reperfusion (IR) injury is a condition in which ischemic myocardium is restituted to normal perfusion after a period of coronary artery occlusion. It has been documented that oxidative stress plays a vital role in myocardial damage caused by myocardial I/R (Cheng et al., 2022). In a rat model of myocardial IR, 50 and 100 mg/kg of carvacrol for 7 days exerted cardioprotective through attenuating infarct size, enhancing SOD and CAT activity, decreasing MDA concentration, and reducing cardiomyocyte apoptosis. This protective effect of carvacrol was associated with the activation of the MAPK/ERK and Akt/eNOS intracellular signal pathways (Chen et al., 2017). The findings of Song et al. (2013), also detected that 60 mg/kg of carvacrol applied cardioprotective against myocardial IR in mice by suppressing oxidative stress and apoptosis. In a rat model of acute MI also 25, 50, and 100 mg/kg of carvacrol for 7 days mitigated the infarct size and lowered the level of creatine kinase (CK), lactate dehydrogenase (LDH), and cardiac troponin T. Inconsistent with the finding of this study, carvacrol diminished MDA concentration and elevated SOD, GSH, and glutathione peroxidase (Yu et al., 2013). The effects of *Z. multiflora* and its main ingredient, carvacrol on LPS‐induced aortic and cardiac injury showed that oral administration of carvacrol (25–100 mg/kg) significantly enhanced the levels of SOD, CAT, and thiols, while decreasing MDA and NO in cardiac and aortic tissues of LPS‐injected rats (Hosseini et al., 2023). Similarly, the effect of carvacrol on LPS‐induced heart dysfunction in Balb/C mice improved the survival rate in mice. Carvacrol also improved the echocardiographic factors and improved the LPS‐induced decline in the ejection fraction (%) and restored the myocardial antioxidants and histopathological changes as well as reduced the levels of proinflammatory mediators in the heart. In addition, carvacrol reduced the protein levels of NLRP3 inflammasome, apoptosis‐associated speck‐like protein (ASC), IL‐18, and IL‐1β in the heart tissue (Joshi et al., 2023).

**FIGURE 2:**
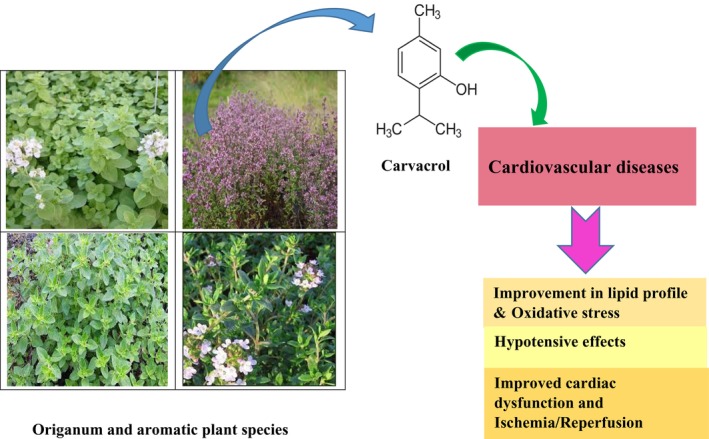
Pharmacological activities of carvacrol on CVDs.

These studies indicated that oxidative stress plays a vital role in myocardial damage caused by myocardial I/R and carvacrol due to the inhibition of inflammatory signaling pathway and ROS production acted as beneficial effects on cardiac dysfunction. Cardioprotective effects of carvacrol on cardiac dysfunction and IR injury are summarized in Table 3. The possible pharmacological activities of carvacrol on CVDs are shown in Figure 2.

**TABLE 3:** Cardioprotective effects of carvacrol on cardiac dysfunction and I/R injury.

Study design	Doses	Route of administration	Effects	Ref.
H9c2 cell	0.01 and 0.1 μM	Expose	↓ The size of rat cardiomyocyte cells. Mitigate the number of apoptotic cells Improved oxidative stress	Jamhiri et al. (2019)
Cardiomyocytes	6.25 and 50 μM	Expose	↓ TLR4/NFκB/NALP3/IL‐1β inflammatory signaling pathway and ↓ ROS production	Marconi et al. (2022)
H9c2 cardiomyoblast cells	2.5, 5, and 10 μg/mL		↓ ROS generation and abates pyroptosis mediated by NOD‐like receptor family pyrin domain containing 3 (NLRP3) inflammasome	Joshi et al. (2023)
Mice model of DM	20 mg/kg	P.O	↓ DCM via modulating PI3K/AKT signaling	Hou et al. (2019)
Diabetic db/db mice	5 and 10 mg/kg	P.O	Improvement of endothelial dysfunction in diabetic db/db mice ↓ The inflammation reactions	Zhao et al. (2020)
Rats	50 and 75 mg/kg	I.P	↓ The level of MDA and expression of ANP ↑ DPPH radical scavenging activity	Jamhiri et al. (2019)
Rats	10, 25, and 50 mg/kg	I.P	Antihypertensive and antiapoptotic effect	Sadeghzadeh et al. (2018)
Rats	50 mg/kg	P.O	Improvement of electrocardiogram recording, troponin I content, and ventricular function	Jafarinezhad et al. (2019)
Rats	25 mg/kg	P.O	↓ Inflammation ↓ Apoptosis Modulation of oxidative stress	El‐Sayed et al. (2016)
Rats	5 and 10 mg/kg	I.P	↓ Inflammation ↓ Lipid peroxidation ↑ Glutathione ↑ Total antioxidant capacity	Cetik et al. (2015)
Rats	50 and 100 mg/kg	I.P	↓ Infarct size ↑ SOD and CAT activity, ↓ MDA concentration ↓ Cardiomyocyte apoptosis Activation of the MAPK/ERK and Akt/eNOS intracellular signaling pathways	Chen et al. (2017)
Mice	60 mg/kg	‐	Suppression of oxidative stress and apoptosis	Song et al. (2013)
Rats	25, 50, and 100	I.P	Mitigation of the infarct size ↓ The level of CK, LDH ↓ Cardiac troponin T ↓ MDA concentration ↑ SOD, GSH, and glutathione peroxidase	Yu et al. (2013)
Rats	25–100 mg/kg	P.O	↑ The levels of SOD, CAT, and thiols ↓ MDA and NO in cardiac and aortic tissues of LPS‐injected rats	Hosseini et al. (2023)
Balb/C mice	‐	‐	↑ Survival rate in induced septic in mice. Improved the echocardiographic parameters ↑ Ejection fraction (%) and fraction shortening (%) Restored the myocardial antioxidants and histopathological changes ↓ The levels of proinflammatory mediators ↓ The protein levels of NLRP3 inflammasome, apoptosis‐associated speck‐like protein (ASC), IL‐18, and IL‐1β	Joshi et al. (2023)

Abbreviations: ANP, Atrial natriuretic peptide; CAT, Catalase; DCM, Diabetic cardiomyopathy; DM, Diabetes mellitus; DPPH, 2‐2‐diphenyl 1‐picril‐hydrasil; I.P, Intraperitoneal injection; I/R, Ischemia/reperfusion; LAD, Left anterior descending coronary artery; MDA, Malondialdehyde; P.O, Per Os or by mouth/oral; SOD, Superoxide dismutase.

## 4 CONCLUSION

Carvacrol as phenolic monoterpene has various pharmacological effects. The results of reviewed studies (in vitro and in vivo) indicated the possible therapeutic effect of carvacrol on CVDs, including lowering BP, modulating lipid profile, improving cardiac dysfunction and ischemic reperfusion (IR) injury, myocardial infarction as well as reducing cardiac toxicity.

The preventive effects of carvacrol on cardiovascular disorders might be linked to its antioxidative, anti‐inflammatory (reduced proinflammatory and enhanced anti‐inflammatory mediator), and antiapoptotic properties. Although the potent effects of carvacrol on CVD were shown in the reviewed studies, further studies such as meta‐analyzing the previous study and clinical trials are needed to be performed.

## AUTHOR CONTRIBUTIONS

**Mohammad Reza Khazdair:** Conceptualization (lead); data curation (lead); methodology (lead); writing – original draft (lead); writing – review and editing (lead). **Mozhgan Moshtagh:** Conceptualization (equal); data curation (equal); methodology (equal); writing – original draft (equal). **Akbar Anaeigoudari:** Conceptualization (equal); data curation (equal); methodology (equal); writing – original draft (equal). **Shima Jafari:** Conceptualization (equal); data curation (equal); methodology (equal); writing – original draft (equal). **Toba Kazemi:** Data curation (equal); writing – review and editing (equal).

## CONFLICT OF INTEREST STATEMENT

The authors declare no conflicts of interest.

## Data Availability

Data sharing is not applicable to this article as no new data were created or analyzed in this study.
